# Socioeconomic Status and Poor Health Outcome at 10 Years of Follow-Up in the Multi-Ethnic Study of Atherosclerosis

**DOI:** 10.1371/journal.pone.0165651

**Published:** 2016-11-22

**Authors:** Steven Shea, Joao Lima, Ana Diez-Roux, Neal W. Jorgensen, Robyn L. McClelland

**Affiliations:** 1 Department of Medicine, Columbia University College of Physicians & Surgeons, New York City, New York, United States of America; 2 Department of Epidemiology, Mailman School of Public Health, Columbia University, New York City, New York, United States of America; 3 Department of Medicine, Johns Hopkins University, Baltimore, Maryland, United States of America; 4 Office of the Dean, Drexel University School of Public Health, Philadelphia, Pennsylvania, United States of America; 5 Department of Biostatistics, University of Washington, Seattle, Washington, United States of America; Swinburne University of Technology, AUSTRALIA

## Abstract

**Background/Objectives:**

Predictors of healthy aging have not been well-studied using longitudinal data with demographic, clinical, subclinical, and genetic information. The objective was to identify predictors of poor health outcome at 10 years of follow-up in the Multi-Ethnic Study of Atherosclerosis (MESA).

**Design:**

Prospective cohort study.

**Setting:**

Population-based sample from 6 U.S. communities.

**Participants:**

4,355 participants In the MESA Study.

**Measurements:**

Poor health outcome at 10 years of follow-up was defined as having died or having clinical cardiovascular disease, depression, cognitive impairment, chronic obstructive pulmonary disease, or cancer other than non-melanoma skin cancer. Absolute risk regression was used to estimate risk differences in the outcome adjusting for demographic variables, clinical and behavioral risk factors, subclinical cardiovascular disease, and ApoE genotype. Models were weighted to account for selective attrition.

**Results:**

Mean age at 10 years of follow-up was 69.5 years; 1,480 participants had a poor health outcome, 2,157 participants were in good health, and 718 were unknown. Older age, smoking, not taking a statin, hypertension, diabetes, and higher coronary calcium score were associated with higher probability of poor health outcome. After multivariable adjustment, participants in the lowest income and educational categories had 7 to 14% greater absolute risk of poor health outcome at 10 years of follow-up compared to those in the next highest categories of income or education (P = 0.002 for both). Those in the lowest categories of both income and education had 21% greater absolute risk of poor health outcome compared to those in the highest categories of both income and education.

**Conclusions:**

Low income and educational level predict poor health outcome at 10 years of follow-up in an aging cohort, independent of clinical and behavioral risk factors and subclinical cardiovascular disease.

## Introduction

As life expectancy increases throughout the world, the numbers and proportion of the population living to older ages are increasing [1], reflecting both reduction in early life mortality and improvement in life expectancy for older people [2]. A comparison of two Danish cohorts of nonegenerians, born in 1905 and 1915 respectively, found that the more recent cohort performed better on tests of physical and cognitive function, suggesting that more people are living to older age with better overall function [3]. Others in the field of aging have investigated the closely related concepts of frailty, functional status, and their correlates [4]. Related work has focused on multi-morbidity [5] and the burden of disease in the elderly. One challenge in this field has been the lack of longitudinal data, which are important because of the potential in retrospective studies for bias arising from recall and from selective cohort truncation to reach the age of survivorship. A second challenge has been lack of data on a full panoply of relevant predictors including demographic data, risk factors, measures of subclinical disease, clinical data, and genetic factors. Recent work by the US Burden of Disease Collaborators examining years of healthy life lost at the population level confirms the continued importance of ischemic heart disease and stroke as the first and third leading causes of years of life lost to premature mortality. Ischemic heart disease is also the leading cause in the U.S. of disability adjusted life years lost [6]. Thus, cardiovascular risk is of particular importance.

An additional challenge is defining a meaningful endpoint that addresses physical, psychological, and cognitive health that can be assessed based on objective rather than self-reported data. We therefore developed a definition of such an endpoint.

The Multi-Ethnic Study of Atherosclerosis (MESA) is a prospective, longitudinal study with detailed characterization of cardiovascular disease risk factors, measures of subclinical cardiovascular disease together with measures of cognitive function, depression, and other variables of interest including genetic information. We therefore used the rich MESA data set to examine the extent to which a range of biomedical, behavioral, and social variables including income and education predict poor health outcome defined to include physical, psychological, and cognitive health at 10 years of follow-up in a large population-based multi-ethnic cohort.

## Methods

### Study Design and Participants

MESA is a longitudinal population-based study of 6,814 men and women aged 45–85 years, without clinical cardiovascular disease at time of entry, recruited from six U.S. communities (Baltimore, MD; Chicago, IL; Forsyth County, NC; Los Angeles County, CA; northern Manhattan, NY; and St. Paul, MN). Sampling and recruitment procedures have been reported [7]. The baseline exam was conducted between 8/1/00 and 7/30/02. Follow-up at 10 years (MESA exam 5) was 76% (n = 4,655) of those alive. Centrally trained and certified study staff performed all participant measurements. The study was approved by the institutional review boards at all participating centers, specifically the Columbia University Medical Center Institutional Review Board, the Harbor-University of California Los Angeles (UCLA) Research and Education Institute Human Subjects Committee, the Johns Hopkins University School of Medicine Joint Committee on Clinical Investigation, the University of Minnesota Human Research Protection Program, the Northwestern University Social and Behavioral Sciences Institutional Review Board, the UCLA Office of Human Research Protection Program, the University of Vermont Committees on Human Research, the Wake Forest University Health Sciences Office of Research Institutional Review Board, and the University of Washington Human Subjects Division. All participants gave written informed consent.

### Predictor Variables

Questionnaires were used to assess age, gender, race/ethnicity, educational and income levels, occupational information, smoking status, and medication use for diabetes, lipid lowering, and hypertension. Classification of race/ethnicity was based on self-identification using questions based on the U.S. 2000 census questionnaire. Occupation was coded using Census 2000 Occupation Codes and categorized based on NIOSH coding [8]. Health insurance status was determined by questionnaire and coded as yes or no. Physical activity level was assessed by questionnaire as previously described^9^ and minutes of activity were summed for each discrete activity type and multiplied by metabolic equivalent (MET) level in order to calculate met-minute/week [9]. Diet was assessed in MESA using a 127-item food frequency questionnaire based on the food frequency questionnaire that was used in the Insulin Resistance Atherosclerosis Study, which was validated in non-Hispanic white, African American, and Hispanic persons, and further modified to include unique Chinese food and culinary practices [10]. A score based on ten food components was derived to estimate conformity with the Mediterranean dietary pattern[11]. Height and weight were measured, and body mass index (BMI) was computed as kg/m^2^. Blood pressure was measured three times at one minute intervals following a standardized protocol [12]. The mean of the second and third resting systolic and diastolic blood pressures was computed. Hypertension was defined as systolic blood pressure ≥140, diastolic blood pressure ≥90 mmHg, or self-reported high blood pressure and on treatment with medication for hypertension [13]. Diabetes was defined as being on treatment with insulin or oral medication for diabetes or fasting glucose ≥126 mg/dl [14].

#### Laboratory Measurements

Fasting blood specimens were analyzed for serum glucose, C-reactive protein, total cholesterol, high density lipoprotein (HDL) cholesterol, and triglyceride levels. Low density lipoprotein (LDL) cholesterol was calculated in plasma specimens having a triglyceride value <400 mg/dL using the Friedewald formula [15]. Apo E4 allele frequency was determined as previously described [16].

#### Coronary Artery Calcium Score

Calcified plaque in the coronary arteries was assessed using computed tomography (CT) following a standardized scanning protocol [17]. Images were read [18] and calibrated [19] at the Harbor-UCLA Research and Education Institute (Torrance, CA). The mean (across two successive scans per participant at the same exam) Agatston coronary artery calcium (CAC) score was computed [20].

### Outcome Variables

#### Cardiovascular Events

Participants were followed for incident cardiovascular events for a median of 10.2 years from their baseline examinations. In addition to five follow-up MESA examinations, a telephone interviewer contacted each participant every 9 to 12 months to inquire about interim hospital admissions, cardiovascular outpatient diagnoses, and deaths. In order to verify self-reported diagnoses, copies were requested of all death certificates and medical records for all hospitalizations and outpatient cardiovascular diagnoses. Next of kin interviews for out of hospital cardiovascular deaths were obtained [21]. Medical records were obtained for approximately 99% of reported hospitalized cardiovascular events and information on 97% of reported outpatient cardiovascular diagnostic encounters. Follow-up telephone interviews were completed in 90% of living participants.

Trained personnel abstracted medical records suggesting possible cardiovascular events. Two physicians independently reviewed all abstracted medical records for endpoint classification and assignment of incidence dates, using pre-specified criteria. If the reviewing physicians disagreed on the event classification, they adjudicated differences. If disagreements persisted, the full events committee made the final classification. Reviewers classified myocardial infarction as definite, probable or absent, based primarily on combinations of symptoms (e.g., chest pain), ECG abnormalities, and cardiac biomarker levels. Coronary heart disease (CHD) death was classified as present or absent based on hospital records and interviews with families. Definite fatal CHD required an MI within 28 days of death, chest pain within the 72 hours before death, or a history of CHD and the absence of a known non-atherosclerotic or non-cardiac cause of death. Adjudicators determined presence of angina based on documentation of chest pain or anginal equivalent together with objective evidence of reversible myocardial ischemia or obstructive coronary artery disease (e.g., ≥70% coronary artery obstruction or positive stress test). Coronary artery revascularization was determined from follow-up interviews and medical records. Stroke required documented focal neurologic deficit lasting 24 hours or until death, or if < 24 hours, presence of a clinically relevant lesion on brain imaging. Patients with focal neurologic deficits secondary to brain trauma, tumor, infection, or other non-vascular cause were excluded. Definite and probable congestive heart failure (CHF) required clinical symptoms (e.g., shortness of breath) or signs (e.g., edema); asymptomatic disease was not an endpoint. Probable CHF further required a physician diagnosis of CHF and medical treatment for CHF. Definite CHF also required pulmonary edema/congestion by chest X-ray and/or dilated ventricle or poor left ventricular function by echocardiography or ventriculography, or evidence of left ventricular diastolic dysfunction. Incident peripheral vascular disease was based on a documented physician diagnosis plus at least one other criteria, namely ultrasound evidence of obstruction; an exercise test positive for claudication; revascularization for PAD; amputation for ischemia; ankle-arm ratio ≤0.8; imaging of an aortic aneurysm; or a vascular procedure for abdominal aortic aneurysm.

### Depression and Cognitive Impairment or Dementia

Depression was assessed using the 20 item Centers for Epidemiological Studies Depression Scale (CES-D) [22] administered at MESA exams 1, 3, 4, and 5. We used the exam 5 CES-D score as part of the assessment of poor health outcome at exam 5. A score of 16 or greater is indicative of mild or significant depression [23]. Cognitive function was assessed at exam 5 only, using the Cognitive Abilities Screening Instrument (CASI) [24]. The CASI assesses performance in multiple domains, including attention, concentration, orientation, short- and long-term memory, language abilities, visual construction, list generating fluency, abstraction, and judgment. Individuals with impairment in one domain do not necessarily have impairments in other domains; thus, a specific score may indicate different impairments in different individuals. It has a range of 0 to 100. We used a cut point of <74 to indicate cognitive impairment [25]. Dementia was ascertained from ICD-9 codes from hospital records whenever a participant was hospitalized and the records were obtained.

#### Chronic Obstructive Pulmonary Disease (COPD) and non-Melanoma Skin Cancer

COPD was determined from ICD-9 codes whenever a participant was hospitalized and the records were obtained. Cancer incidence was based on self-report by participants at telephone or in person follow-up.

#### Poor Health Outcome at 10 Years of Follow-up

Poor health outcome at 10 years of follow-up was defined as meeting any of the following criteria at MESA exam 5: (a) occurrence of death; (b) occurrence of incident CVD (CHD, stroke, congestive heart failure, peripheral vascular disease); (c) presence of depression, defined as score 16 or greater on the CES-D; (d) presence of cognitive impairment, defined as a score of <74 on the CASI, or of dementia; (e) presence of chronic obstructive pulmonary disease; and (f) occurrence of cancer other than non-melanoma skin cancer. The comparison group (“In good health at 10 years of follow-up”) comprised those alive at exam 5 and having none of the criteria for “poor health outcome”. Those lost to follow-up, who did not attend Exam 5, or who attended exam 5 but did not complete either the CASI or the CES-D were classified as “Unknown”, unless they had evidence of a prior event.

### Timing of Measurements

All predictor variables were measures at baseline (MESA exam 1). Occurrence of death, incident CVD, COPD, dementia, and cancer were ascertained continuously over the follow-up time between MESA exam 1 and exam 5 as described above. Depression and cognitive impairment were ascertained at MESA exam 5 using questionnaires, as described above.

### Statistical Analysis

Characteristics of participants with known health status (either unhealthy or healthy) differed from those with unknown health status. We therefore used an inverse probability weighting approach to address this selection bias. For each participant, the probability of having unknown health status at the follow-up exam was modeled using logistic regression as a function of the baseline covariates listed in Table 1. Each participant with known health status (either unhealthy or healthy) was weighted by the inverse of the probability that the participant was non-missing. Robust standard errors were used to account for the impact of the probability weights on the standard errors. Bivariate comparisons and multivariable analysis were performed using the weighted sample. Multivariable absolute risk regression analysis was performed with all covariates in the model. These models estimate differences in absolute risk or probability between categories of participants for the outcome of interest. Inclusion of all relevant covariates in the fully adjusted model did not violate assumptions related to collinearity. Effect modification between income and education was tested by including the interaction term in the model. The impact of the interaction was illustrated graphically by displaying average predicted risks at each level of income and education jointly.

**Table 1:** Balance of Baseline Characteristics by Unknown Health Status at 10 Years of Follow Up in MESA Before and After Inverse Probability Weighting (IPW).

	Health Status at 10 Years of Follow Up					
	Before Weighting			IPW		
Baseline Characteristics	Known	Unknown	p-value	Known^1^	Unknown	p-value
	(N = 3637)	(N = 718)		(N = 3637)	(N = 718)	
	Mean (SD) or N (%)	Mean (SD) or N (%)		Mean (SD) or N (%)	Mean (SD) or N (%)	
Age, y	61.3 (9.9)	62.7 (10.7)	0.001	61.5 (9.9)	61.4 (10.7)	0.774
Male	1916 (52.7)	358 (49.9)	0.167	1897 (52.2)	370 (51.4)	0.716
Race/ethnicity						
White	1531 (42.1)	217 (30.2)	<0.001	1460 (40.2)	286 (39.7)	0.810
Chinese	466 (12.8)	124 (17.3)		490 (13.5)	91 (12.7)	
Black	944 (26.0)	192 (26.7)		951 (26.2)	198 (27.6)	
Hispanic	696 (19.1)	185 (25.8)		735 (20.2)	144 (20.0)	
Married	2339 (64.3)	442 (61.6)	0.161	2320 (63.8)	452 (62.8)	0.628
Income						
<$25K	913 (25.1)	277 (38.6)	<0.001	992 (27.3)	194 (26.9)	0.961
$25K-$49K	1046 (28.8)	202 (28.1)		1044 (28.7)	214 (29.8)	
$50K-$99K	1091 (30.0)	160 (22.3)		1045 (28.7)	204 (28.3)	
$100K+	587 (16.1)	79 (11.0)		556 (15.3)	108 (15.0)	
Education						
< High school	488 (13.4)	168 (23.4)	<0.001	545 (15.0)	104 (14.5)	0.908
High school	1663 (45.7)	323 (45.0)		1661 (45.7)	339 (47.2)	
College	710 (19.5)	125 (17.4)		697 (19.2)	133 (18.6)	
Graduate school	776 (21.3)	102 (14.2)		733 (20.2)	142 (19.8)	
Occupation						
Professional	1711 (47.0)	280 (39.0)	0.002	1661 (45.7)	321 (44.7)	0.887
Service	507 (13.9)	135 (18.8)		533 (14.7)	113 (15.8)	
Sales/Office	747 (20.5)	156 (21.7)		746 (20.5)	154 (21.5)	
Construction	210 (5.8)	38 (5.3)		213 (5.9)	40 (5.6)	
Production	462 (12.7)	109 (15.2)		484 (13.3)	90 (12.5)	
Smoking Status						
Never	1828 (50.3)	376 (52.4)	0.340	1841 (50.6)	371 (51.5)	0.903
Former	1366 (37.6)	249 (34.7)		1347 (37.0)	260 (36.2)	
Current	443 (12.2)	93 (13.0)		448 (12.3)	89 (12.3)	
No Health Insurance	247 (6.8)	103 (14.4)	<0.001	290 (8.0)	55 (7.7)	0.726
Intentional Exercise						
(met-min/week)	1621 (2358)	1658 (2308)	0.699	1630 (2414)	1675 (2182)	0.624
Mediterranean Diet						
0–3	1119 (30.8)	232 (32.3)	0.130	1128 (31.0)	226 (31.4)	0.976
4	615 (16.9)	139 (19.4)		629 (17.3)	122 (16.9)	
5	693 (19.1)	132 (18.4)		689 (18.9)	139 (19.3)	
6	564 (15.5)	113 (15.7)		567 (15.6)	116 (16.1)	
7–10	646 (17.8)	102 (14.2)		624 (17.2)	117 (16.2)	
BMI	28.1 (5.4)	28.2 (5.5)	0.909	28.1 (5.4)	28.2 (5.3)	0.984
Diabetes	382 (10.5)	87 (12.1)	0.203	391 (10.8)	76 (10.6)	0.917
Hypertension	1533 (42.2)	317 (44.2)	0.322	1545 (42.5)	308 (42.8)	0.884
Hypertension Meds	1290 (35.5)	254 (35.4)	0.962	1289 (35.5)	253 (35.2)	0.915
Systolic BP, mm Hg	125.1 (20.7)	126.8 (21.2)	0.053	125.4 (20.8)	125.3 (20.2)	0.880
Diastolic BP, mm Hg	72.2 (10.3)	71.9 (9.4)	0.473	72.1 (10.3)	72.1 (9.3)	0.997
Statin Meds	547 (15.0)	99 (13.8)	0.389	538 (14.8)	99 (13.8)	0.529
Total Cholesterol,mg/dL	193.4 (34.3)	194.6 (35.5)	0.389	193.6 (34.5)	193.6 (35.7)	0.984
LDL Cholesterol, mg/dL	117.4 (31.0)	119.0 (32.3)	0.217	117.7 (31.1)	117.8 (32.5)	0.922
HDL Cholesterol, mg/dL	51.0 (14.7)	49.9 (13.9)	0.052	50.8 (14.6)	51.1 (15.1)	0.699
Ln(CRP)	0.6 (1.1)	0.6 (1.2)	0.538	0.6 (1.1)	0.6 (1.2)	0.910
Apo E4						
None	2643 (72.7)	537 (74.8)	0.304	2654 (73.0)	523 (72.7)	0.965
Heterozygote	908 (25.0)	161 (22.4)		893 (54.6)	177 (24.7)	
Homozygote	86 (2.4)	20 (2.8)		88.9 (2.4)	19 (2.6)	
CAC: ln(CAC+1)	2.2 (2.5)	2.2 (2.5)	0.929	2.2 (2.5)	2.2 (2.5)	0.923

^1^ Note: Known/Unknown sample sizes are the sample sizes after weighting in the IPW columns.

MESA, Multi-Ethnic Study of Atherosclerosis. BMI, body mass index. LDL, low density lipoprotein. HDL, high density lipoprotein. CAC, coronary artery calcium Agatston score. CRP, C-reactive protein.

## Results

**Fig 1:**
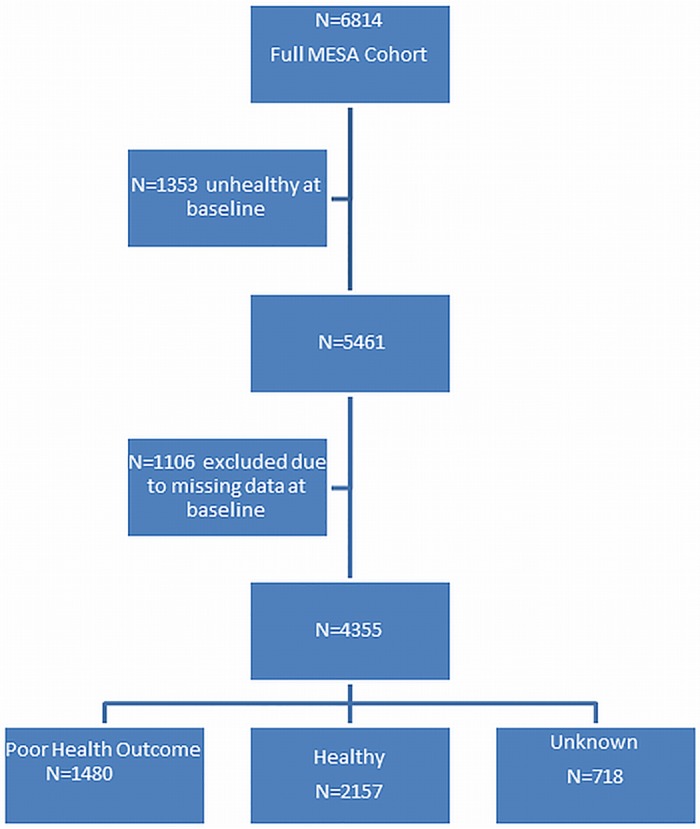
Flow chart of participant exclusions from the study. Definition of Poor Health Outcome at MESA Exam 5: Occurrence of death, cancer (excluding non-melanoma skin cancer),chronic obstructive pulmonary disease (COPD), cardiovascular disease (CVD; defined as myocardial infarction (MI), cardiac arrest, stroke, congestive heart failure (CHF), or peripheral vascular disease (PVD), cardiac revascularization, or definite angina), or presence of cognitive impairment or depression at MESA exam 5. Definition of Healthy: None of the above diseases/conditions.

Of 6,814 participants at MESA exam 1, 1,353 were excluded because they met criteria for poor health outcome at Exam 1 and an additional 1,106 were excluded because relevant baseline data were missing. At exam 5, 1,480 had a poor health outcome, 2,157 were in good health, and 718 were unknown (Fig 1). The mean age of the cohort was 61.3 years at the baseline exam and 69.5 years at exam 5. The differences between those with known and unknown health status at exam 5 were large prior to weighting, with the potential for substantial bias. After inverse probability weighting, these differences were greatly reduced and all were non-significant (Table 1).

In bivariate analysis (Table 2), baseline factors that predicted poor health outcome at 10 years of follow up included older age, male sex, black or Hispanic race/ethnicity (with white as reference group), smoking, presence of diabetes or hypertension, higher systolic and diastolic blood pressure, higher BMI, lower HDL cholesterol, higher coronary artery calcium score, higher CRP level, lower family income, lower educational attainment, occupation, and being unmarried.

**Table 2:** Weighted Summary and Bivariate Comparison of Baseline Characteristics by Health Outome Status at 10 Years of Follow Up in MESA.

Baseline Characteristics	Poor Health Outcome^1^	Healthy	p-value
	(N = 1480)	(N = 2157)	
	Mean (SD) or N (%)	Mean (SD) or N (%)	
Age, y	**64.8 (9.8)**	**59.2 (9.2)**	**<0.001**
Male	**879 (58.6)**	**1018 (47.7)**	**<0.001**
Race/ethnicity			
White	**574 (38.3)**	**886 (41.5)**	**<0.001**
Chinese	**172 (11.5)**	**318 (14.9)**	
Black	**417 (27.8)**	**535 (25.0)**	
Hispanic	**337 (22.4)**	**398 (18.6)**	
Married	**889 (59.2)**	**1431 (67.0)**	**<0.001**
Income			
<$25K	**553 (36.8)**	**439 (20.6)**	**<0.001**
$25K-$49K	**434 (28.9)**	**610 (0.29)**	
$50K-$99K	**356 (23.7)**	**689 (32.3)**	
$100K+	**158 (10.6)**	**398 (18.6)**	
Education			
< High school	**321 (21.4)**	**225 (10.5)**	**<0.001**
High school	**686 (45.7)**	**975 (45.7)**	
College	**242 (16.1)**	**456 (21.3)**	
Graduate school	**525 (16.8)**	**481 (22.5)**	
Occupation			
Professional	**601 (40.0)**	**1060 (49.6)**	**<0.001**
Service	**262 (17.5)**	**271 (12.7)**	
Sales/Office	**285 (19.0)**	**461 (21.6)**	
Construction	**119 (7.9)**	**94.4 (4.4)**	
Production	**234 (15.6)**	**250 (11.7)**	
Smoking Status			
Never	**685 (45.7)**	**1156 (54.1)**	**<0.001**
Former	**583 (38.9)**	**764 (35.8)**	
Current	**232 (15.5)**	**216 (10.1)**	
No Health Insurance	**115 (7.7)**	**175 (8.2)**	**0.372**
Intentional Exercise (met-min/week)	**1548 (2314)**	**1688 (2480)**	**0.091**
Mediterranean Diet			
0–3	**468 (31.2)**	**661 (30.9)**	**0.615**
4	**276 (18.4)**	**352 (16.5)**	
5	**279 (18.6)**	**410 (19.2)**	
6	**227 (15.2)**	**340 (15.9)**	
7–10	**251 (16.7)**	**374 (17.5)**	
BMI	**28.6 (5.4)**	**27.9 (5.4)**	**<0.001**
Diabetes	**233 (15.5)**	**158 (7.4)**	**<0.001**
Hypertension	**774 (51.6)**	**771 (36.1)**	**<0.001**
Hypertension Meds	**664 (44.2)**	**625 (29.3)**	**<0.001**
Systolic BP, mm Hg	**129.3 (21.6)**	**122.7 (19.8)**	**<0.001**
Diastolic BP, mm Hg	**72.7 (10.6)**	**71.7 (10.1)**	**0.005**
Statin Meds	**251 (16.7)**	**288 (13.5)**	**0.006**
Total Cholesterol, mg/dL	**191.8 (33.7)**	**194.9 (34.9)**	**0.010**
LDL Cholesterol, mg/dL	**116.7 (30.7)**	**118.3 (31.3)**	**0.138**
HDL Cholesterol, mg/dL	**49.4 (14.2)**	**51.8 (14.8)**	**<0.001**
Ln(CRP)	**0.7 (1.1)**	**0.5 (1.1)**	**<0.001**
Apo E4			
None	**1073 (71.5)**	**1581 (74.0)**	**0.143**
Heterozygote	**384 (25.6)**	**509 (23.8)**	
Homozygote	**43.4 (2.9)**	**45 (2.1)**	
Agatston CAC: ln(CAC+1)	**3.0 (2.7)**	**1.6 (2.2)**	**<0.001**

^1^ Note: Poor Health Outcome/Healthy sample sizes are the sample sizes after weighting. Sample sizes may not add up to column totals due to rounding.

MESA, Multi-Ethnic Study of Atherosclerosis; BMI, body mass index. LDL, low density lipoprotein. HDL, high density lipoprotein. CAC, coronary artery calcium Agatston score. CRP, C-reactive protein.

**Fig 2:**
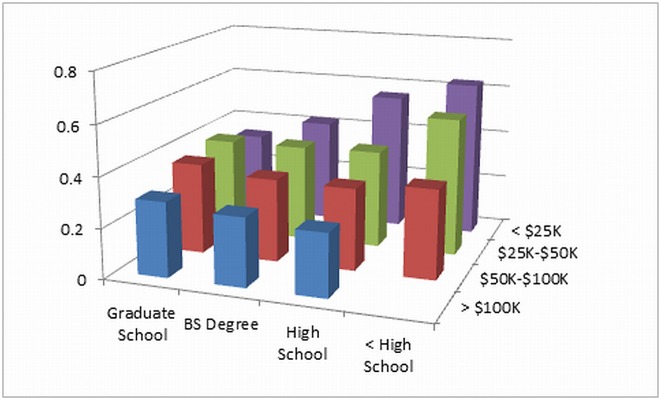
Joint probabilities (vertical axis) of poor health outcome at 10 years of follow up in MESA by income (family income per year) and education level.

In multivariable analysis (Table 3), after adjustment for all predictor variables, those in the lowest family income group (<$25,000/year) had 7% greater probability of poor health outcome at 10 years of follow-up compared to those in the next highest income group and 14% greater probability compared to those in the highest income group. Those in the lowest education group (< high school) had 8% greater probability of poor health outcome at 10 years of follow-up compared to those in the next highest education group, with similar differences between other education groups. Older age, male gender, current smoking, hypertension, diabetes, and higher calcium score were also significantly associated with higher probability of poor health outcome at 10 years of follow-up. Variables that were not statistically significant predictors in the fully adjusted model included marital status, no health insurance, physical activity level, Mediterranean diet score, and body mass index. Compared to those in the lowest category of both income and education, those at the next highest level of both income and education had 7% greater risk, and those at the highest level of both had 21% greater risk, of poor health outcome at 10 years of follow up. The interaction between family income and educational level was statistically significant (p < 0.001) (Fig 2), such that the association of education with poor health outcome at Exam 5 was stronger at the lowest level of family income than at higher levels, and vice versa.

**Table 3:** Weighted Multivariable Absolute Risk Regression for Poor Health Outcome At 10 Years of Follow Up in MESA.

Probability of Poor Health Outcome		
Model Terms^1^ (N = 3637)	β [95% CI]	p-value
Age^2^	0.09 [0.07,0.11]	<0.001
Gender		
Female	ref	
Male	0.07 [0.04,0.11]	<0.001
Race/ethnicity		
White	ref	
Chinese	-0.06 [-0.11,-0.00]	0.034
Black	-0.03 [-0.07,0.01]	0.129
Hispanic	-0.04 [-0.09,0.01]	0.111
Married	-0.02 [-0.06,0.01]	0.246
Income		
<$25K	ref	
$25K-$49K	-0.07 [-0.12,-0.03]	0.002
$50K-$99K	-0.11 [-0.16,-0.06]	<0.001
$100K+	-0.14 [-0.20,-0.07]	<0.001
Education		
< High school	ref	
High school	-0.08 [-0.14,-0.03]	0.002
College	-0.09 [-0.15,-0.02]	0.007
Graduate school	-0.08 [-0.15,-0.02]	0.015
Occupation		
Professional	ref	
Service	0.05 [-0.01,0.10]	0.092
Sales/Office	-0.01 [-0.06,0.03]	0.516
Construction	0.07 [-0.00,0.15]	0.056
Production	0.004 [-0.05,0.06]	0.980
Smoking Status		
Never	ref	
Former	0.01 [-0.02,0.04]	0.550
Current	0.14 [0.09,0.19]	<0.001
No Health Insurance	-0.004 [-0.07,0.06]	0.915
Intentional Exercise, met-min/week	-0.01 [-0.03,0.00]	0.073
Mediterranean Diet		
0–3	ref	
4	-0.005 [-0.05,0.04]	0.834
5	-0.01 [-0.05,0.04]	0.798
6	-0.01 [-0.06,0.04]	0.734
7–10	-0.004 [-0.05,0.04]	0.856
BMI	0.02 [-0.00,0.04]	0.076
Diabetes	0.09 [0.03,0.14]	0.001
Hypertension Meds	0.06 [0.02,0.09]	0.001
Systolic BP, mm Hg	0.01 [-0.01,0.03]	0.301
Statin Meds	-0.04 [-0.08,0.01]	0.094
Total Cholesterol, mg/dL	-0.01 [-0.03,0.00]	0.068
HDL Cholesterol, mg/dL	-0.01 [-0.02,0.01]	0.435
Ln(CRP)	0.02 [-0.00,0.03]	0.064
Apo E4		
None	ref	
Heterozygote	0.04[0.00,0.07]	0.045
Homozygote	0.10 [0.00,0.20]	0.048
Agatston CAC: ln(CAC+1)	0.06 [0.04,0.08]	<0.001

^1^ All variables included from Table 1 other than hypertension and LDL Cholesterol; hypertension was omitted because both systolic blood pressure level and use of anti-hypertensive medications were included in the model. LDL was omitted because total and HDL cholesterol were included in the model.

^2^ Continuous variable estimates are per standard deviation.

MESA, Multi-Ethnic Study of Atherosclerosis. BMI, body mass index. HDL, high density lipoprotein. CAC, coronary artery calcium Agatston score. CRP, C-reactive protein.

There were numerically important contributions from each of the component endpoints to having a poor health outcome at 10 years of follow-up (Table 4). The associations of lower family income and lower educational level were statistically significant for all components of the outcome other than cancer.

**Table 4:** Weighted Summary of Baseline Characteristics by Event for Participants with Poor Health Outcome at 10 Years of Follow Up in MESA.

Baseline Characteristics	Unhealthy^1^	Death	CVD^2^	Depression	Cognitive Impairment	COPD	Cancer
	N = 1480	N = 379	N = 495	N = 329	N = 265	N = 93	N = 499
	N (row %)	N (row %)	N (row %)	N (row %)	N (row %)	N (row %)	N (row %)
Female	**621 (35.7)**†	**140 (8.1)**†	**171 (9.8)**†	**161 (9.3)**	**129 (7.4)**	**37 (2.2)**	**175 (10.1)**†
Male	**879 (46.3)**	**239 (12.6)**	**324 (17.1)**	**168 (8.9)**	**136 (7.2)**	**55 (2.9)**	**303 (16.0)**
Race/ethnicity							
White	**574 (39.3)**†	**137 (9.4)**†	**209 (14.3)**†	**129 (8.8)**	**53 (3.6)**†	**31 (2.2)**	**219 (15.0)**†
Chinese	**172 (35.2)**	**40 (8.1)**	**47 (9.6)**	**44 (8.9)**	**28 (5.7)**	**13 (2.7)**	**39 (7.9)**
Black	**417 (43.8)**	**126 (13.3)**	**125 (13.2)**	**75 (7.8)**	**72 (7.6)**	**34 (3.6)**	**150 (15.8)**
Hispanic	**337 (45.8)**	**76 (10.3)**	**114 (15.5)**	**83 (11.3)**	**112 (15.2)**	**14 (1.9)**	**71 (9.6)**
Income							
<$25K	**553 (55.7)**†	**179 (18.1)**†	**170 (17.1)**†	**104 (10.5)**†	**149 (15.0)**†	**48 (4.8)**†	**142 (14.3)**
$25K-$49K	**434 (41.5)**	**95 (9.1)**	**164 (15.7)**	**106 (10.2)**	**73 (7.0)**	**19 (1.8)**	**126 (12.1)**
$50K-$99K	**356 (34.0)**	**79 (7.6)**	**110 (10.6)**	**80 (7.7)**	**39 (3.7)**	**21 (2.1)**	**144 (13.8)**
$100K+	**158 (28.5)**	**25 (4.6)**	**51 (9.1)**	**40 (7.2)**	**5 (0.9)**	**5 (0.8)**	**66 (11.9)**
Education							
< High school	**320 (58.8)**†	**82 (15.1)**†	**98 (18.0)**†	**67 (12.2)** †	**112 (20.5)**†	**24 (4.5)**†	**71 (13.1)**
High school	**686 (41.3)**	**183 (11.0)**	**232 (14.0)**	**147 (8.9)**	**111 (6.7)**	**43 (2.6)**	**211 (12.7)**
College	**242 (34.7)**	**62 (9.0)**	**79 (11.3)**	**57 (8.2)**	**25 (3.5)**	**14 (2.0)**	**90 (12.9)**
Graduate school	**252 (34.4)**	**52 (7.0)**	**86 (11.7)**	**58 (7.9)**	**18 (2.5)**	**12 (1.6)**	**107 (14.6)**
Smoking Status							
Never	**685 (37.2)**†	**155 (8.4)**†	**204 (11.1)**†	**153 (8.3)**†	**163 (9.0)**†	**17 (1.0)**†	**199 (10.8)**†
Former	**583 (43.3)**	**155 (11.5)**	**203 (15.1)**	**121 (9.0)**	**77 (5.7)**	**43 (3.2)**	**212 (15.7)**
Current	**232 (51.9)**	**69 (15.4)**	**87 (19.5)**	**55 (12.2)**	**25 (5.5)**	**32 (7.1)**	**67 (15.1)**

^1^ Notes: Sample sizes are the sample sizes after weighting. Sample sizes may not add up to column totals due to rounding. Row total is less than sum of numbers in row because some participants had more than one condition as of exam 5.

^2^ MESA, Multi-Ethnic Study of Atherosclerosis.

CVD Endpoint: MI, cardiac arrest, stroke, CHF, PVD, revascularization, definite angina.

^†^ Statistically significant at p = 0.05.

## Discussion

The main finding of this study is that MESA participants at the lowest income and educational categories had 7 to14% greater absolute risk of poor health outcome at 10 years of follow-up. This disadvantage was observed in the lowest income and education groups, namely those reporting family income less than $25,000 per year in 2000–2002 and those with less than high school education, compared to the next highest income and education groups. There was additional disadvantage compared to the highest family income group, while the education groups other than the lowest were similar. Those in the lowest categories of both income and education had 21% higher absolute risk of a poor health outcome compared to those in the highest categories of both income and education. These findings were apparent in bivariate analyses and also after adjustment for demographic variables, clinical and behavioral risk factors, and measures of subclinical cardiovascular disease including coronary calcium score. Several other baseline characteristics, including older age, smoking, hypertension, diabetes, and higher coronary calcium score were also, as expected, associated with greater risk of a poor health outcome at 10 years of follow-up. The associations of low income and low education with the specific health outcomes that contributed to poor health outcome at 10 years of follow-up were consistent for each of the components, other than cancer. Of note, with the exception of slightly lower probability of poor health in Chinese participants, race/ethnicity was not significantly associated with poor health after other variables were controlled.

Longitudinal studies of older people of necessity need to have been started years ago and sustained over a long period. Most longitudinal studies have focused on risk factors for specific diseases, or on functional status, with only a few that have specifically addressed healthy aging. For example, studies have been done of people who have reached 100 with relative vigor, but these studies have necessarily had to collect data retrospectively. Some of these studies have focused on genetic associations, in part as a way to avoid this problem [26]. An exception is the work of Vaillant et al. [27–29] who studied a cohort of Harvard men recruited in the 1940s and whose surviving members are still being followed. This work has focused on psychological adjustment, mental health, and alcoholism. Other relevant work includes that of Newman et al., who published a comparison of the Long Life Family Cohort (LLFC), who were sampled conditional in part on living to age 79 or greater in relatively good health, to individuals in three other cohorts including the Framingham Heart Study, The Cardiovascular Health Study, and the Framingham Offspring Study [30]. Not surprisingly, the LLFC has lower prevalence of diabetes, vascular disease, and chronic obstructive pulmonary disease, and higher levels of measures of functional capacity and performance. Diehr et al. applied a predictive model in data from the Cardiovascular Health Study to estimate future years of healthy life, where the definition of healthy life was based on the response to the question “Is your health excellent, very good, good, fair, or poor?” [31] but these authors did not test specific risk factors or analyze an objective measure of the outcome. The American Heart Association, as part of its effort to promote cardiovascular health, issued a report on behalf of its Strategic Planning Task Force and Statistics Committee in which it defined the concept of *cardiovascular health* (or *ideal cardiovascular health*) as presence of four favorable health behaviors (nonsmoking, body mass index <25 kg/m^2^, physical activity at goal level, diet consistent with guidelines), presence of four favorable health factors (abstinence from smoking within the last year, untreated total cholesterol <200 mg/dl, untreated blood pressure <120/80 mmHg, fasting blood glucose <100 mg/dl), and absence of clinical CVD [32]. However, other than absence of CVD, these are risk factors, not states of health or well-being. The Cardiovascular Health Study (CHS) All Stars Study conducted an examination in 2005–06 of CHS participants aged 77 years and older that included measure of cognitive and physical function [33]. Several publications from CHS All Stars are relevant [34,35,36,37,38,39,40] but none specifically addressed the predictors of reaching older age in good physical, cognitive, and psychological health.

Our findings were inconsistent with respect to whether there is a gradient across categories of income and education, depending on the socioeconomic measure. We found such a gradient for family income but not for educational attainment (Table 3). This pattern may be contrasted with the graded association with civil service class and health outcomes seen in the Whitehall Study [41]. This difference may reflect the different social circumstances in the U.S. compared to Great Britain as well as the fully employed, working age sample in the Whitehall Study, which differs from MESA. We did find statistically significant evidence of an interaction between income and educational level in our data (Fig 2). The interaction observed suggests a synergistic effect of lower income and lower education (adverse effect of low income is potentiated by low education and vice versa).

We considered several factors that could potentially have contributed to the associations we observed between low income and education levels with poor health outcome at 10 years of follow-up. One such factor is lack of access to regular health care, however lack of health insurance was not a significant predictor of the outcome in either bivariate or fully adjusted analyses. We also considered physical activity level and diet, but these variables also were not significant in either bivariate or fully adjusted analyses. Chetty et al. recently reported an association between higher income and greater life expectancy in the U.S. [42] Similar to our findings, Chetty et al. did not find that access to health care correlated with the outcome. Other potential explanatory variables examined by Chetty et al. included local labor markets, residential segregation, and adverse effects of inequality as measured by the Gini index, but their analyses did not find support for any of these [42] These investigators inferred that most of the variation in life expectancy was explained by differences in smoking, physical activity, and obesity, however we adjusted for all of these variables and still found important differences by income and education level in the probability of poor health outcome at 10 years of follow-up in MESA. Other work in this area has explored the roles of stress and allostatic load [43,44] and of social context [45,46], which includes physical, psychological, and social aspects of the environment, as potential factors mediating the relationship between socioeconomic status and health outcomes.

Several limitations are noted. First, the use of a composite endpoint has potential drawbacks, in that subjects can attain or fail to attain the endpoint through multiple pathways. This non-specificity is potentially offset by the clinical and psychological salience of an endpoint that broadly indexes healthy aging. Non-composite endpoints may also occur through multiple pathways. This endpoint is innovative in that is derived from the data available in MESA as a best definition of overall health, but to our knowledge there are no prior publications using this outcome definition. Secondly, the use of the CASI to define cognitive impairment is not fully defined based on prior literature. In addition, the CASI was not administered at all exams. Third, the cancer component of the outcome was based on self-report. Fourth, we used family income rather than wealth to classify socioeconomic disadvantage. However, the classification of individuals to income groups used in our analysis is unlikely to change substantially based on income and wealth data combined. Strengths include the longitudinal design of the study, the high rate of follow-up, the multi-ethnic and population-based sample, the uniquely well characterized cardiovascular risk factors and subclinical cardiovascular disease measures in MESA, use of standardized measures of cognitive function and depression, availability of Apo E genetic data, and ascertainment of both cardiovascular and non-cardiovascular disease endpoints.

It might be argued that diabetes should be included in the criteria for poor health outcome, whereas we included diabetes mellitus among the predictors of poor health outcome at 10 years of follow-up. We did so because not including diabetes in the set of predictors or covariates would have increased the potential for confounding. We also note that for cardiovascular risk factors, diabetes has traditionally been considered as a predictor [47]. Diabetes is also a predictor of depression [48] and cognitive impairment [49,50]. We acknowledge that the presence of diabetes may be considered by some to be a poor health outcome.

In summary, we found that lower family income and lower educational attainment independently predict poor health outcome at 10 years of follow-up in the MESA study. These differences were of substantial magnitude, ranging from 7 to 14% in absolute risk difference compared to the next highest categories of income and education, and 21% difference in risk for those in the lowest categories of both family income and education compared to those in the highest categories of both. These findings illustrate the impact of low income and education level on failing to remain in good health at older age. The unfavorable impact included adjustment for clinical factors and was observed across a range of components of the poor health metric, suggesting that clinically targeted interventions alone may not be sufficient to address preventable and costly loss of health in the aging population.
